# Systematic review of non-surgical treatments for early dupuytren’s disease

**DOI:** 10.1186/s12891-016-1200-y

**Published:** 2016-08-15

**Authors:** Catherine Ball, David Izadi, Liaquat Suleman Verjee, James Chan, Jagdeep Nanchahal

**Affiliations:** Kennedy Institute of Rheumatology, University of Oxford, Roosevelt Drive, Headington, Oxford, OX3 7FY UK

**Keywords:** Dupuytren’s disease, Systematic review, Pharmacological therapy, Radiotherapy, Physical therapy

## Abstract

**Background:**

Dupuytren’s disease is a common fibrotic disorder of the palm characterized by the development of progressive flexion deformities in the digits, leading to significant functional impairment. Surgical excision remains the most common treatment. However, this is only indicated in patients with established contractures rather than those with early disease. Early disease is generally characterized by the presence of palmar nodules with limited or no contracture of the fingers. The ideal treatment would be directed at patients with early progressive disease to prevent future deterioration. Various non-surgical treatment modalities have been described but there is currently no systematic assessment of the role and efficacy of these treatments in patients with early disease.

**Methods:**

Using a PICOS analysis we reviewed publications of studies of patients with early disease who had received physical therapies, pharmacological treatment, or radiotherapy. Following PRISMA guidelines titles and abstract were screened using predefined criteria to identify those reporting outcomes specifically relating to the treatment of early disease. In the absence of a definition of early disease studies were included if early DD was described clinically, with digital contractures not exceeding 30°, Tubiana grades N to 1, and which reported identifiable data. Studies were excluded if data for early DD patients could not be extracted for analysis.

**Results:**

In this systematic review, 26 studies were identified and analyzed to evaluate the effect of pharmacological therapy (*n* = 11), physical therapy (*n* = 5) and radiotherapy (*n* = 10) on early Dupuytren’s disease. The studies comprised 20 case series, 1 cohort study with the remainder reporting case studies. All publications were graded level of evidence 4 or 5 assessed using the Oxford Centre for Evidence Based Medicine grading. Narrative descriptions of the data are presented.

**Conclusions:**

Physical therapies were the most robustly assessed, using objective measures but the studies were under powered, providing insufficient evidence of efficacy. Intralesional steroid injection and radiotherapy appeared to lead to softening of nodules and to retard disease progression but lacked rigorous evaluation and studies were poorly designed. There is an urgent need for adequately powered double blinded randomized trials for this common disorder which affects 4 % of the population.

**Trial registration:**

The protocol was registered (CRD42015008986 16 November 2015) with the PROSPERO international prospective register of systematic reviews.

**Electronic supplementary material:**

The online version of this article (doi:10.1186/s12891-016-1200-y) contains supplementary material, which is available to authorized users.

## Background

Dupuytren’s disease (DD) is a common fibroproliferative disorder of the hand affecting approximately 4 % of the general UK and US populations [1, 2]. The prevalence of DD in the general population increases with age and in a recent systematic review was estimated as 12 % in those aged 55 years, rising to 29 % in those aged 75 years in the general population in western countries [3]. The classic description of disease progression is the initial appearance of nodules, with subsequent formation of cords. This is followed by a final stage as the cords mature and irreversible digital contractures develop, resulting in significant impairment of hand function [4]. However disease progression is not inevitable, with only 30-50 % going on to develop progressive flexion deformities [5, 6] and that the course of DD may fluctuate over time [7].

One of the earliest classifications of Dupuytren’s disease according to the histological appearance using optical microscopy [8] described 3 stages: proliferative, involutional and residual. Further sophistication was added by correlating histological appearance with the clinical findings and ultrastructural features [4]. This was extended by Lam [9] using electron microscopy to include the relative proportion of type III collagen based on the finding that earlier lesions have a higher proportion of type III collagen, which changes to a greater proportion of type I collagen at later stages of the disease.

One of the few groups to study tissues collected at all clinical stages of the disease also classified the disorder into 3 stages [10]:

I. Early disease. Specimens comprised nodules from patients with no digital contracture. These showed proliferating spindle shaped cells surrounded by fine granulofibrillary material although there was no increased collagen deposition in the nodule.
II. Active disease. Clinically these patients presented with palmar thickening and associated joint contracture, with the contracture noted by the patient as occurring on average over 3 years. The nodules comprised mainly of myofibroblasts, with very little intervening collagen. The nodules were associated with cords, which were relatively acellular.
III. Advanced disease. These patients had progressive joint contracture for more than 3 years. Microscopic examination revealed relatively few cells that were elongated and embedded in stroma comprising a large amount of mature collagen fibers.

A study of surgically excised specimens from patients with digits flexed to 30° or greater and with functional impairment of the hand showed that even in this group nodules comprising aggregates mainly of myofibroblasts with interspersed inflammatory cells are embedded within the cords and anatomically lie adjacent to the flexed joint [11]. Furthermore, patients with more advanced deformities were less likely to have identifiable nodules, corresponding to the advanced stage described by Chiu et al. [10].

A number of clinical grading and staging systems that record the presence of palmar nodules, cords and the degree of digital flexion to reflect disease severity have been proposed [8, 10, 12–19] (Table 1).

**Table 1:** Clinical staging and grading systems for Dupuytren’s disease

Author	Iselin (1951) [13]	Shaw (1951) [57]	Steinberg (1951) [18]	Luck (1959) [8]	Early (1962) [12]	Chiu and McFarlane (1978) [10]	Tubiana (1968) [17]	Tubiana: Keilholz modification (1996) [14]	Tubiana: Seegenschmiedt modification (2001) [15]
Grade or Stage	**0**: Small nodules, hand function not affected	**1**: Hands with a nodule in the palmar fascia not yet involving the skin and causing no contracture of the fingers	**1**: Fibrosis of palmar fascia without contractures	**Proliferative stage**: nodules in the palm and fingers	**0**: Palmar nodules	**Early disease**: nodules in the palmar fascia without digital contracture	**N**: Palmar nodule without presence of contracture	As Tubiana	As Tubiana
	**1**: Nodules and cords in the palm and early contracture of MCPJ	**2**: Nodule in the fascia involving the skin but not causing finger flexion deformity	**2**: Contractures up to 135° toward the palm	**Involutional stage**: development of finger flexion contractures	***1 point***: *total digital deformity of* ≤*60*°	**Active disease**: nodular thickening with associated digital contracture	**1**: Total flexion deformity (TFD) between 0° and 45°	**N**/**1**: TFD 1–5	**N**/**1**: TFD 1–10
	**2**: MCPJ contracture up to 30° and early PIPJ contracture	***3***: *Nodule in the palm invading skin plus flexion contracture of one or more fingers*	***3***: *Contractures up to 90*° *toward the palm*	***Residual stage***: *cord development and finger contractures with joint changes*	***2 points***: *61 to 120*°	***Advanced disease***: *Progressive joint contracture for more than 3 years with diffuse palmar fibrotic thickening*	***2***: *TFD between 45*° *and 90*°	**1**: TFD 6–45	**1**: TFD 11–45
	***3***: *Contracture of IPJs more than 30*°	***4***: *Includes all stage 3 cases in which the secondary changes have occurred in the tendons or joints of one or more fingers*.	***4***: *Fibrosis of palmar fascia*, *with flexion deformities of the fingers beyond 90*° *toward the palm*.		***3 points***: >*120*		***3***: *TFD between 90*° *and 135*°	*As Tubiana*	*As Tubiana*
	***4***: *Extreme digital flexion contracture. Sensory and circulatory disturbance*						***4***: *TFD greater than 135*°	*As Tubiana*	*As Tubiana*

Normal text indicates early stage disease as defined for this review. Italic text indicates later stage disease not included in this review

Currently there is no formal clinical definition of early disease and no widely accepted treatment. Expert opinion suggests that non-surgical treatments for DD are not effective in reversing or retarding disease progression [20] and are generally considered ineffective [21]. However, the evidence has not been systematically evaluated [22].

The mainstay of treatment for patients with established flexion deformities is surgery. Surgery is considered if a finger has lost 30° of metacarpophalangeal joint or any proximal interphalangeal joint extension [23]. Surgery is not advocated in early DD except when it is associated with persistent pain, especially at night or in the rare circumstances where a trigger finger requiring surgical release necessitates access to the A1 pulley deep to a DD nodule [24]. Furthermore, surgery undertaken during the more cellular, proliferative stage of the disease is considered to be associated with a higher rate of recurrence [25].

## Methods

A systematic review was performed to determine the role and efficacy of non-surgical treatments for early DD and to provide an evidence base for the management of these patients. The search strategy and search terms were based on a Participants, Intervention, Comparison, Outcomes and Study (PICOS) design [26] (Additional file 1). A literature search was performed using controlled subject headings and free text terms for “Dupuytren’s disease” and “non-surgical therapies”. A broad range of terms covering pharmacological therapies, radiotherapy and physical therapies were used to formulate a comprehensive and inclusive search strategy for non-surgical therapies for early DD. The protocol was registered (CRD42015008986) with the PROSPERO international prospective register of systematic reviews [27].

### Inclusion and exclusion criteria

Studies evaluating non-surgical treatment of adults with early DD where outcomes were monitored using patient reported outcome measures, physical measures, clinical assessment and clinical observation were included. Randomized and non-randomized controlled clinical trials, prospective and retrospective case series, case studies, conference abstracts and letters were eligible for inclusion. Studies comprising all stages of DD were scrutinized to extract data pertaining to early DD where possible. Early disease was defined as a baseline contracture of 30° or less at each affected digital joint or as a grade or description of palmar involvement with digital contracture of 30° or less.

Studies involving 2 or more digits on 1 hand were excluded if any digital contracture exceeded 30° indicating more advanced disease in the hand. Studies reporting treatment of later stage DD, recurrent DD or postoperative DD were excluded. Patients within studies who had received treatment previously for DD in the pertinent hand were excluded. There was no language restriction for eligibility for inclusion. There was no restriction regarding duration of post intervention monitoring (Additional file 2).

### Search methods and identification of studies

**Fig. 1:**
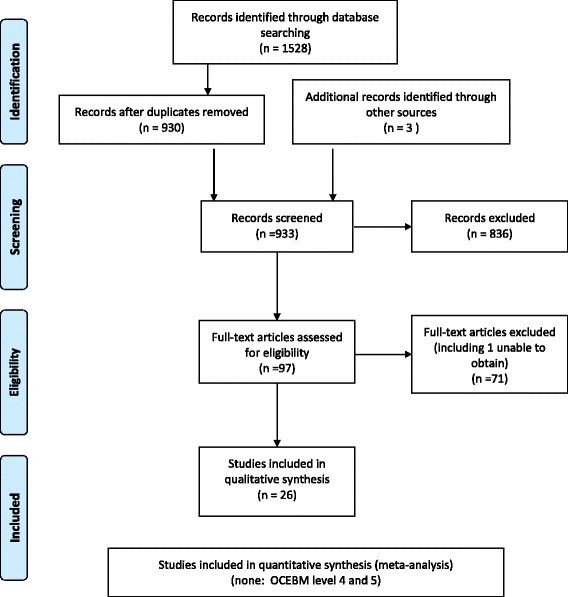
PRISMA flow diagram

Ovid Medline and Embase databases were searched from inception to October 2015. A total of 930 references, following removal of duplicates by both electronic and manual screening, were downloaded into a bibliographic software package (EndNote ×7). Three additional studies were identified from a personal bibliography. In total 97 studies were identified by title/abstract review applying the eligibility criteria after independent review by 2 authors (CB and DI or LV), resulting in 26 studies meeting the inclusion criteria (Fig. 1).

Where necessary, consensus was achieved by reviewing the full text by a fourth author (JN). Full texts of all studies were further reviewed by the same 2 authors (CB and DI or LV) to identify data specifically relating to early DD (Additional file 3).

### Data collection and analysis

Data on study design, intervention, study dates, criteria used to identify early DD, number of early DD patients, number of total cohort, outcomes measured, baseline data and results were collected and tabulated on an Excel (Microsoft, Seattle) spreadsheet. Adverse events when reported were noted. Due to the heterogeneity of the studies and variability in the techniques used to analyse and report the data, pooled analysis or meta-analysis were not warranted and narrative descriptions of the data are presented. The quality of studies was assessed using the Oxford Centre for Evidence Based Medicine (OCEBM) levels of evidence criteria [28]. Risk of bias was assessed using GRADE guidelines, Cochrane Handbook for Systematic Reviews [29].

## Results

Of the 26 studies that met the inclusion criteria, 11 pertained to pharmacological treatment [18, 30–39], 5 to physical therapies [40–44] and 10 to radiotherapy [14, 45–53]. All included studies were observational and graded OCEBM level 4 or 5. The risk of bias for all studies was high. Three studies declared no conflict of interest [42, 51] or financial gain [31], one study declared grant funding from the Squibb Institute of Medical Research [18] and one study disclosed that cortisone was supplied by the National Research Council of Canada [30].

The studies comprised 20 case series [14, 18, 31, 33, 34, 36, 37, 40–42, 44–53], 6 case studies [30, 32, 35, 39, 43] with 2 by 1 author [30], and 1 cohort study [38]. Only 4 publications described results for cohorts comprising exclusively of early DD patients [31, 46, 48, 49]. The remaining studies included early DD patients within cohorts of patients with more advanced disease.

### Pharmacological therapy

Eleven studies related to pharmacological therapies (Table 2), including steroids [30–34], vitamin E [18, 35, 36], furazolidone injection [37], aminosyn [38] and hyperbaric oxygen [39].

**Table 2:** Summary of results of pharmacological treatment

Author (year) treatment	Total cohort of DD patients (hands)	Number of patients (hands) with early DD	Study type	Level of evidence (OCEBM) Prospective (P) Retrospective (R) Not stated (N)	Outcome measure	Results			Recurrence	Adverse events
						Improved	No change	Deteriorated		
Steroids										
Baxter (1952) [30], Intramuscular	11 (16)	1 (2)	Case study	5 (N)	Clinical observation of nodules, extension deficit	0	1	0	Not reported	Not reported
Oral		1 (2)	Case study	5 (N)	Clinical observation of palmar fascia	0	1	0	Not reported	Not reported
Ketchum (2000) [31], Injection	63 (75)	63 (75)	Case series	4 (N)	Nodule easier to inject and flatter	62 patients (73 hands)	0	1 patient (2 hands)	50 % of patients at 1 to 3 years	50 % patients reported transient depigmentation or temporary subcutaneous atrophy at injection site. ‘Nearly all’ resolved at 6 months
					Clinically observed digital contracture	0	62 patients (73 hands)	1 patient (2 hands)		
Shelley (1993) [32], Topical	2	1	Case study	5 (N)	Contracture shrinkage	1	0	0	None at 2 years	Not reported
Zachariae (1955) [33], Injection	11 (11)	9 (9)	Case series	4 (N)	Fibrosis diminished or softer	9	0	0	1 at 14 months	Not reported
Coste (1953) [34], Injection	9 (13)	2 (2)	Case series	4 (N)	Clinical observation of nodules, extension deficit	2	0	0	Not reported	Not reported
Vitamin E (oral)										
Reilly (1974) [35]	1 (2 hands)	1 (2 hands)	Case study	5 (N)	Clinical observation	0	0	1 patient (2 hands)	Eventually required surgery in both hands	Not reported
Richards (1952) [36]	70 hands	63 hands	Case series	4 (N)	Observation, deformity monitored using plaster cast moulds	0	60	3	Not reported	No toxic effects
Steinberg (1951) [18]	22	6	Case series	4 (N)	Clinical observation	6	0	0	Not reported	Not reported
Other										
Skliarenko (1982) [37], furazolidone injection	98	74	Case series	4 (N)	Clinically observed digital contracture	74	0	0	9 patients in total cohort (4 at 1 year, 3 at 2 years, 2 after 3 years)	18 of total cohort of 98 reported minor hand swelling, raised temperature after 1st injection. No systemic side effects.
Gatev (1997) [38], Topical aminosyn and ultrasound	63	22	Cohort study	4 (N)	Clinical observation of palm thickening, tension and trembling	21	1	0	Not reported	Not reported
Topical aminosyn		4				4	0	0		
Ultrasound		6				4	2	0		
Yildiz (2004) [39], Hyperbaric oxygen	1	1	Case study	5 (N)	Extension deficit, clinical observation	1	0	0	None at 1 year	No adverse effects.

Summary of results of pharmacological treatment from each study, including the number of patients with Dupuytren’s disease in the total cohort in each study, the number of patients with early disease within the total cohort, study type and design, level of evidence according to the Oxford Centre for Evidence Based Medicine (OCEBM) criteria, the outcome measure used and results. The number of hands (in brackets) is stated when available, with recurrence and adverse events where stated

### Steroids

#### Intralesional steroid injection

Three studies reported the use of intranodular [31], intralesional [33] or immediately below the nodule [34] steroid injection. In an early study [34] 2 cases of early DD defined as Meyerding Stage 0 within a larger cohort of DD patients were administered hydrocortisone injections ‘immediately below the nodule’. Each was injected with 5 and 12mgs respectively once a week for 4 weeks. Outcomes were assessed clinically as defined by ‘nodule disappearance’ and results for both cases described as ‘good’. The follow-up period was not stated.

The rationale for intranodular and intralesional steroid injections was based on early clinical and experimental studies examining the inhibitory effect on connective tissue development [33], and subsequently on degradation of mature collagen in hypertrophic scars [31]. All reported clinically observed measures as outcomes.

Zachariae [33] injected hydrocortisone acetate in 9 patients (9 hands) with early DD as defined by palmar fibrosis with no contracture, or a total contracture of all joints of a digit of 30° or less. Six patients received a total of 3 injections of 25 mg, 1 patient 2 injections of 50 mg, 1 patient received 2 injections of 10 mg followed by 1 injection of 25 mg, and 1 patient was injected twice with 25 mg. All injections were administered at 2–3 weekly intervals over a 2–5 week period. The follow-up period ranged from 2 to 24 months. Outcome was assessed clinically and fibrosis reported subjectively to be ‘diminished’, ‘slightly diminished’ or ‘softened’ in all cases, with resolution of pain in both cases describing pain at baseline. Recurrence after 14 months was reported in the patient who received two 10 mg and one 25 mg injection.

A larger and more recent case series comprised a retrospective review of 63 patients (75 hands) with early DD defined as a flexion contracture of less than 15° at the metacarpophalangeal (MCP) joint and no contracture at the proximal interphalangeal (PIP) joint at baseline [31]. All patients were treated using a series of intranodular injections with triamcinolone acetonide. The dose per patient ranged between 80-120 mg at each visit at 6 weekly intervals and an average of 3.2 injections at each site. After a period of 6 months, 3 further injections were given if required. The follow-up period ranged from 30 months to 27 years. The outcome measure used was clinician rated ease of injection. Seventy three hands (62 patients) were reported as having nodules described ‘easier to inject’ and to show 60–80 % regression as defined by the nodules being ‘flatter’. There was no change in digital contracture in this group. However, the remaining patient with bilateral disease required surgery. Disease reactivation requiring one or more further injections was observed in 50 % of patients 1 to 3 years after the last injection. Adverse events were reported for 50 % of patients, including transient depigmentation or subcutaneous atrophy at the injection site, all of which were described as having resolved within 6 months of the last injection.

#### Topical steroids

Topical application of steroid cream has been reported to suppress local immunological inflammatory change [54]. One patient with early DD, defined as a painful fibrous cord with no restriction of digital extension, was treated with topical application of clobetasol cream twice daily and 0.1 % tretinoin cream once daily [32]. At 3 months, pain had resolved and the contracture was described as ‘shrinking’. By 9 months the hand was reported as ‘normal’ and there was no recurrence at 2 years.

#### Oral steroids

There is only 1 case report of a patient treated with oral steroids for early DD. The patient was described as having bilateral early DD with restriction of digital extension of the little and ring fingers of the left hand by 1 and 2 centimeters respectively, and minimal changes with no extension restriction in the right hand. He was treated with a total oral dose of 200 mg cortisone acetate daily for 3 weeks but there was no clinically observable change [30].

#### Intramuscular steroids

A patient with bilateral early DD defined by lack of full digital extension by 0.5 cm treated with 200 mg of cortisone daily intramuscularly for 2 weeks also showed no improvement [30].

### Vitamin E

Three studies reported use of vitamin E [18, 35, 36]. Based on clinical findings that daily administration of 200 mg of ephynal for 20 weeks resulted in a decrease in palmar fibrosis and an increase in digital extension in 13 patients (22 hands) affected to varying degrees by DD and who had not previously had surgery [55], it was hypothesized [18] that vitamin E downregulates fibroblast activity. Six patients with early DD defined as grade 1 (fibrosis of the palmar fascia without contractures), were treated with 300 mg of mixed natural tocopherols for up to 7 months. Four patients were reported to be ‘cured’ and 2 ‘improved’ [18].

A study of 63 hands [36] with early DD defined as thickening of the palmar fascia only or digital flexion deformity of 30° or less at either the MCP or interphalangeal joints reported on the efficacy of systematic vitamin E. Oral doses of 100 mg of tocopherol acetate were administered twice daily for a minimum of 3 months. No rationale for treatment was described. Plaster cast molds were used to record the deformity at baseline and compared with the presenting deformity at follow up. No clinically observable improvement was reported in 60 hands, and deterioration occurred in 3 hands. Toxic effects of treatment were not seen. In a case study Reilly [35] described developing early DD with a small hard nodule in his right palm, with subsequent digital flexion, and similarly in the left hand 3 years later. He reported self-administration of 400 mg oral alpha tocopherol succinate daily for 14 years with no effect on either of his hands, eventually requiring surgery for both hands.

### Other

A cohort study [38] applied topical aminosyn with (*n* = 22) or without ulltraphonophoresis (ultrasound) (*n* = 6), or ultrasound only (*n* = 4) to patients with early DD defined as palmar thickening and tension, pain and trembling being grade 1 on a grading scale of 1 to 3. An application of 1–2 centimeter thickness aminosyn with or without ultrasound, or ultrasound alone was administered 5 days a week for 3–4 min for a total of 12-18 procedures. Outcomes were measured by clinical examination and patient reported softening and reduced thickening of contractures at the end of treatment and were categorized in 5 classifications from ‘clinically healthy’ to ‘no improvement’. Across all groups, 29 of the 32 patients improved and 3 showed no change.

Furazolidon (20 mg) was injected into the affected palmar aponeurosis of 74 patients with early disease [37]. Treatment was stated to be based on the anti-fibrotic effect of the drug. Early disease was defined as Stages 1 and 2 according to the presence of nodules and cords with digital joint contractures up to 30°. Patients were given a total of 5–7 injections at 10–14 day intervals. Outcomes were defined by clinically observed reduction of contracture and softened nodules and cords and categorized as ‘good’, ‘satisfactory’ or unsatisfactory’. On examination at 1–5 years after injection, 70 patients were classified as ‘good’ and 4 as ‘satisfactory’. Of the total cohort of 98 patients, 18 reported minor hand swelling and raised temperature after the first injection. No systemic toxic effects were found.

Hyperbaric oxygen was administered to a single patient with early disease described as an isolated palmar cord with no nodule and 10° MCP joint extension deficit [39]. The authors based the treatment on the premise that high tissue oxygenation could reverse local ischemia that they postulated could be associated with DD. Following 40 sessions totaling 60 h of treatment over 2 months, the palmar cord was reported to be no longer visible and full extension of the joint was achieved, with no adverse effects of treatment.

### Physical therapy

Five studies reported physical therapy for the treatment for early DD, including ultrasound [40], splinting [41, 42], frictional massage [43] and heat treatment with joint stretching [44] (Table 3).

**Table 3:** Summary of results of physical therapy treatment

Author (year) treatment	Total cohort of DD patients (hands)	Number of patients (hands) with early DD	Study type	Level of evidence (OCEBM) Prospective (P) Retrospective (R) Not stated (N)	Outcome measure	Results			Recurrence	Adverse events
						Improved	No change	Deteriorated		
Markham (1980) [40], Ultrasound	8 (9 hands)	3 (4 hands)	Case series	4 (P)	Digital joint extension in degrees (*n* = 4)	2	(2 had no extension deficit at baseline)	0	None	Not reported
					Clinical observation (*n* = 2)	1 patient (2 hands)				
					Hand span (*n* = 4)	2	2			
					Grip strength (*n* = 4)	4				
Ball (2002) [41], Splinting	6 (7 hands)	5 (6 hands)	Case series	4 (P)	Active digital joint extension in degrees	4 patients (5 hands)	1 patient (1 hand)	0	None at 2 years	Not reported
Larocerie-Salgado, (2012) [42], Splinting	13 (13 hands)	2	Case series	4 (P)	Digital joint extension in degrees	2 patients	0	0	Not reported	Not reported
Christie (2012) [43], Frictional massage, (Treatment hand)	1 (2 hands)	1 (2 hands)	Case study	5 (P)	Active digital joint extension in degrees	1 hand (2 digits)	0	0	Not reported	Not reported
(Control hand)						0	0	1 hand (2 digits)		
Onat (2013) [44], Heat, splinting, stretching	3	2	Case series	5 (N)	Degrees of digital motion	2	0	0	Not reported	Not reported

Summary of results of physical therapy treatment for each study, including the number of patients with Dupuytren’s disease in the total cohort in each study, the number of patients with early disease within the total cohort, study type and design, and level of evidence according to the Oxford Centre for Evidence Based Medicine (OCEBM) criteria, the outcome measure used and results. The number of hands (in brackets) is stated when available, with recurrence and adverse events where stated

A series of 3 patients (4 hands, 6 digits) with early DD [40] from a larger cohort of 8 patients were treated using low intensity therapeutic ultrasound combined with physical mobilization and joint stretching. The rationale for use was based on the premise that treatment with ultrasound leads to softening and increased extensibility of the fibrous tissue. Outcomes were assessed using objective physical measures and clinical examination. Active digital joint extension was measured in degrees using a goniometer, hand span in centimeters, power grip strength was measured in pounds, and palmar consistency assessed by clinical examination. Patients were treated with ultrasound for between 4 and 10 min weekly over 5 to 8 weeks until no further improvement was observed. Exercise and stretching followed each ultrasound treatment. Two patients with limitations in digital extension (4 fingers) were reported as having improved between 5 to 23°. Palmar tissue in both hands of a patient with palmar thickening and no digital limitation was considered to have become softer. Hand span increased in 2 patients by 0.7 and 2.4 centimeters and remained unchanged in both hands of the third patient. Grip strength improved in all 3 patients by between 3 and 6.5 pounds.

Two studies reported on the use of night splints with patients with early DD [41, 42], with outcomes comparing the degree of active individual digital joint extension measured using a goniometer before and after treatment. The studies were based on the premise that low load tension promotes tissue remodeling [42] as a result of increased matrix metalloproteinase activity [56]. A prospective study of 5 patients (6 digits) with early DD treated with thermoplastic palmar based finger extension splints worn at night reported increased active digital extension in 4 patients (5 digits) and no change in 1 patient. Improvement ranged from 2 to 12° at follow-up between 4 and 24 months from baseline [41]. A more recent study reported the results of 2 early DD patients with PIP joint contractures in a larger cohort of 13 DD patients [42]. Both patients treated using a combination of extension splinting, stretching exercises and friction massage improved by 20° at follow-up at 5 and 22 months respectively. Two patients in a study of physical therapy received paraffin bath heat treatment combined with joint stretches, ultrasound and splinting [44]. Early disease was defined by degrees of digital extension. Following 15 therapy sessions improvements of 10° were reported for both patients.

A case study of a patient with early DD compared the results of treating the ring and little fingers of 1 hand using frictional massage and stretching, with the contralateral hand treated using stretching alone [43]. Early DD was identified by the presence of palmar nodules in both hands with a cord of DD in 1 hand but no flexion deformity; however, the ability to actively hyperextend the affected digits was limited in both hands. Treatment was based on the supposition that cross-frictional massage may soften contractile structures, although the exact mechanism is subject to debate. Each hand was treated with 2 min of stretching with or without additional friction massage 3 times weekly for 8 weeks. Active digital hyperextension was measured in millimeters. Increases of 1 mm and 13 mm were reported at 4 months in the ring and little fingers, respectively, in the combined massage and stretching treatment hand as compared to baseline. In contrast, decreases of 2 mm and 3 mm respectively were reported at 4 months in the hand treated by stretching alone. Comparison with pre-treatment photographs showed a decrease in contracted palmar skin of the left hand. At 8 weeks there was no change noted in the area of the nodules as assessed by ultrasound scan.

### Radiotherapy

Ten publications reporting outcomes of patients with early DD treated with radiotherapy met the inclusion criteria, six from Germany [14, 45–49] with the remainder from Italy [50], Australia [51] and 2 from the UK by the same author [52, 53] (Table 4).

**Table 4:** Summary of results of radiotherapy treatment

Author (year) Treatment	Total cohort of DD patients (hands)	Number of patients (hands) with early DD	Study type	Level of evidence (OCEBM) Prospective (P) Retrospective (R) Not stated (N)	Outcome measure	Results			Recurrence	Adverse events
						Improved	No change	Deteriorated		
Keilholz (1996) [14] Radiotherapy	(142 hands)	(129 hands)	Case series	4 (R)	Clinical assessment of consistency and size of nodule	102	25	2	Not reported	EORTC^a^ Grade 1 and 2 toxicity for total cohort.
Lukacs (1978) [45] Radiotherapy	36	32	Case series	4 (N)	Clinical assessment of softening of nodules, contracture improvement	26	6	0	Not reported	Not reported
Hesselkamp (1981) [46] Radiotherapy	46	46	Case series	4 (N)	Clinical assessment of softening of nodules and cords	24	19	3	Not reported	63 % dry skin with desquamation, 24 % skin atrophy, pigmentation and telangiectasia.
Adamietz (2001) [47] Radiotherapy	99 (176 hands)	(156 hands)	Case series	4 (R)	Tubiana grade	18	79	59, (27 within and 32 outside RT field)	At 10 years >20 % N (*n* = 13), >20 % N/1 (*n* = 13), 65 % stage 1 (*n* = 30.	For total cohort of 176 hands at median 10 years, 44 reported strong desquamation and 15 cutaneous telangiectasia with subcutaneous atrophy.
Kohler (1984) [48] Radiotherapy	29 (33 hands)	29 (33 hands)	Case series	4 (N)	Clinical assessment of softening of DD tissue	7	20	6	1 outside the radiotherapy area.	Not reported
Weinzierl 1993) [49] Radiotherapy *n* = 34	39 (56 hands)	39 (56 hands)	2 Case series	4 (N)	Clinical assessment of consistency and size of nodules	3	14	17	Not reported	32 % had small but ongoing skin change (dry skin).
Injection Superoxide dismutase *n* = 22						7	9	6	Not reported	No local or systemic adverse effects.
Corsi (1966) [50] Radiotherapy, plesiotherapy plus vitamin E	11 (13 hands)	10 (11 hands)	Case series	4 (N)	Clinical assessment of skin consistency, nodule size and digital extension.	8	3	0	Not reported	Temporary skin rash and epidermolysis noted at end of treatment (number affected not given).
Grenfell (2014) [51] Radiotherapy	6 (4 hands)	3 (4 hands)	Case series	4 (N)	Clinical assessment whether nodule size and hardness	4	0	0	None at 34–42 months	Acute side effects: minimal fatigue, mild local oedema and erythema for total cohort. Number affected and duration not given.
Finney (1953) [52] Radiotherapy	25	7	Case series	4 (N)	Clinical assessment of functional improvement	6	1	0	None at 2–10 years	1^st^ degree reaction: skin dryness, slight erythema for total cohort. Number affected not given.
Finney (1955) [53] Radiotherapy	18	3	Case series	4 (N)	Clinical assessment of functional improvement	3	0	0	Not reported	2^nd^ degree reaction: skin dryness, persistent paraesthesia for total cohort. Number affected not given. Paraesthesia persisting up to 12 months in 2 cases.

Summary of results of radiotherapy treatment for each study, including the number of patients with Dupuytren’s disease in the total cohort in each study, the number of patients with early disease within the total cohort, study type and design, level of evidence according to the Oxford Centre for Evidence Based Medicine (OCEBM) criteria, the outcome measure used and results. The number of hands (in brackets) is stated when availabl, with recurrence and adverse events where stated

^a^Toxicity criteria of the Radiation Therapy Oncology Group (RTOG) and the European Organization for Research and Treatment of Cancer (EORTC)

Seven patients with early DD according to Shaw stages 1 and 2 [57] were identified in a study of 25 patients [52]. Radiotherapy treatment was based on the premise that histological changes in DD could be compared to keloid formation so that the mitotic cycle of fibroblasts could be interrupted with radiotherapy, resulting in a reduced matrix deposition. Patients were treated with a total dose of 3000 rads (30 Grays (Gy)) gamma radiation fractioned over 8 days. Outcomes were evaluated by assessment of softening of nodules, reduction in paresthesia and increase in finger movement. Results were presented as 1 of 4 categories indicating the degree of functional improvement ranging from ‘no change’ to ‘full functional recovery’. No data were given for finger movement or detail of clinical assessment of nodules and paresthesia. Full functional recovery was reported in 6 patients and no change in 1 patient. The follow-up period ranged between 2 and 10 years. These results were re-presented along with new data for 3 patients with early disease (Shaw stage 1) in a study of 18 patients treated with medium voltage X-ray treatment over a shorter period in a later publication by the same author [53]. A total of 1500 rads (15 Gy) was administered in 3 fractions over 5 days, with ‘full functional recovery’ reported for 2 patients and ‘functional improvement’ for 1 patient. The method of assessment was not given.

Thirty two patients with early DD defined as having no contractures from a larger cohort of 36 patients were treated with a total dose of between 2400 and 3200 rads, equivalent to 24–32 Gy [45]. Radiation therapy was administered in 2 daily doses of 400r (4 Gy), repeated at 8 week intervals and patients were reassessed to 5 years after treatment. Outcomes were based on the consistency of the nodules. Improvement of 26 of the 32 patients was reported and disease progression arrested in the remaining 6 patients.

Four studies [14, 47, 49, 51] employed the same radiotherapy regime of 3 Gy daily for 5 days, repeated after a 6 week period. Patients were identified with early disease at baseline by modified Tubiana stage N, N/1 and 1 [14, 47], Tubiana stage N,1 [51] or Iselin grade1 [49]. Results were presented as ‘regression’, ‘halted/stable condition’ or ‘progression’ [14, 47, 49] or by describing clinical findings after treatment [51]. Weinzierl [49] reported treatment with radiotherapy of 34 hands. Outcomes were assessed by clinical evaluation of nodule size and consistency at 7 years after treatment. Regression was observed In 3 hands, 14 had no change and 17 progressed. The same author reported a subsequent cohort of 22 hands who received once weekly injections of 8–13 mg of an enzyme, superoxide dismutase, into the affected area for 12 weeks. At 3 years 7 hands regressed, 9 had no change and 6 progressed. A study of 156 hands from a larger cohort of 176 hands in 99 patients [47] reported regression in 18 hands, no change in 79 hands and progression in 59 hands, 27 within and 32 outside the radiotherapy field when followed up at 7 to 18 years after treatment. Results were reported according to disease stage and also assessed by the degree of digital contracture, although data for this outcome were not given.

A retrospective series compared nodule size and consistency before and after radiotherapy in different Tubiana stages [14]. From a larger cohort of 142 hands with DD, 129 hands with early DD graded as N, N/1 or 1 were identified. Two independent clinical assessors evaluated the dimensions of the nodules and their consistency. Finger flexion deformities were measured with a protractor, although data were not given. At 3 months, 10 of 129 hands showed an improvement in grade, and 2 hands were downgraded. Nodule size was reported to have reduced and palpable nodules and cords became softer in 102 of 129 hands. At long term follow-up at a mean of 6 years (range 1 to 12 years), data were only presented for the whole cohort with no sub-analysis of patients with early DD.

A more recent study of a series of 3 patients with early DD was presented in a cohort comprising a further 3 patients with plantar disease [51]. Outcomes were based on the clinical assessment of size and consistency of nodules or lesions, pain or discomfort and restriction of movement. Decrease in nodule size was reported in 1 patient and flatter, softer nodules with reduced discomfort for 1 patient. A reduction in nodule size was reported in the third patient who had bilateral disease but no comment regarding the movement limitation noted at baseline.

Radiotherapy was combined with plesiotherapy, a type of superficial radiotherapy, and vitamin E in a study of 10 patients (11 hands) with Iselin stage 1 and 2 DD [50]. Outcomes were assessed at 6 months to 1 year after treatment by clinical examination of nodule consistency and by DD grade progression. Radiotherapy of 7,500 to 800 rads (7.5 to 8Gy) fractioned in 5 sessions and 1 cycle of plesiotherapy of 2400 or 2500 rads (2.4 to 2.5 Gy) was supplemented by ‘prolonged’ vitamin E although no dose or method of administration was given. Improvement was reported in 8 hands and no change in 3. Radiotherapy totaling 20 Gy fractionated daily or 3 times weekly was administered in 29 patients (33 hands) with Iselin grade 1 DD (Kohler). Seven hands were reported was having improved, ‘no change’ in 20 hands and ‘progression’ in 6 hands, although the assessment of outcome and the follow-up period was not clear. Hesselkamp [46] followed up 36 patients between 1964 and 1979 graded Iselin grade 1 DD at between 1 to 9 years after radiotherapy treatment. Patients received 2 series of 3 to 5 treatments of 400 rads (4 Gy). Results were categorized as ‘better’ (24 patients), ‘no change’ (19 patients and ‘worse’ 3 patients based on clinical examination of softening of nodules and cords.

Adverse events were reported by 8 of the 10 radiotherapy studies, with 2 reporting early DD cases [46, 49] and 6 reporting for total cohorts [14, 47, 50–53]. In 34 patients with early DD, ongoing dry skin was seen at 7 year follow-up in 32 % [49] and at 1–9 year follow-up of 46 patients in 62 % [46], with the latter also reporting skin atrophy, depigmentation and telangiectasia in 24 % of patients. Grade 1 or ‘mild’ reactions were reported in 3 studies [50–52] although numbers affected were not given. More severe 2^nd^ degree reactions with severe paresthesia were reported as “the rule” in 1 study of medium voltage X-ray treatment [53] persisting in 2 of 34 patients for up to a year before resolving. Keilholz [14] reported grade 1 acute mild skin reactions in 43 % and grade 2 radiodermatitis in 9.8 % of the total cohort of 142 hands, with ‘most patients’ describing itching and burning sensations during treatment. Within the irradiated area minor long term radiogenic skin and subcutaneous changes were seen in 77 % hands, comprising 64 % with dry skin and increased desquamation and 13 % mild skin atrophy with ‘slight fibrosis’ or occasional telangiectasia. At a median of 10 years following treatment dry skin with strong desquamation was reported in 44 (25 %) of 170 hands and subcutaneous atrophy in 15 (8.5 %) hands [47].

## Discussion

There are a range of treatments available for patients with advanced DD with digital contractures, including surgical excision of the diseased tissue, percutaneous needle fasciotomy and collagenase injection. However they all suffer from disadvantages. The ideal would be a treatment that prevents progression in the 30–50 % [5, 6] of at risk patients with early DD. A variety of treatments have been proposed for early DD but there is currently no clear evidence for their role and efficacy. This systematic review attempts to address this issue. However, it is clear that the evidence base is very weak, with all relevant studies identified as level 4 or 5 rated according to OCEBM and with a high risk of bias so that results of treatment are difficult to interpret. The high risk of bias in observational studies is due to a number of factors, particularly lack of control treatment, retrospective or unclear design and assessor bias, especially in small cohorts of patients. Publication bias, the increased likelihood of authors to write up positive results and of journals to publish positive results introduces further risk.

### Pharmacological therapy

Steroids are known to reduce inflammation which probably precedes all types of fibrosis [58]. Recent studies support the concept of DD as a localized inflammatory disorder [11, 58]. Three studies reported improvement following treatment with intralesional steroid injection [31, 33, 34] using subjective outcomes. The treatment appeared comparatively safe and the reported adverse effects were relatively transient. Within the limitations of the methodology the results would appear to be encouraging. However, there are severe limitations of the assessment methodology in these studies. Objective blinded trials are required before this potentially useful treatment can be recommended. Systemic steroids appear to be of no benefit and there are very limited data for the efficacy of topical application.

The 3 studies examining the effect of vitamin E administered orally gave conflicting results and there is insufficient evidence to support the use of vitamin E for the treatment of early DD.

The favourable outcome reported in the single case report utilizing hyperbaric oxygen is insufficient to support the use of this modality given the substantial clinical resources and clinical burden involved. The positive results of topical aminosyn, an amino acid solution, with ultrasound in 22 patients [38] are confounded by the study also reporting inconclusive results for ultrasound and positive results for aminosyn alone with small numbers of patients. Injection with furazolidone [37] is not recommended as it has been banned from use in humans and animals in the USA and European Union amid concerns as a potential carcinogen.

### Physical therapy

Physical therapy studies were the most robustly assessed, using objective physical measures of digital joint extension [40–44], hand span and grip strength [40]. Although a trend towards digital extension improvement was seen, sample sizes were small and the evidence to support the use of physical therapies very limited and inconclusive.

One study attempted to objectively evaluate changes in subcutaneous features of DD using ultrasound imaging to monitor treatment outcome, but failed to detect change at 8 weeks following treatment [43]. Ultrasound imaging has been used to identify DD in the hand [59] but has not been used to examine change over longer periods and research to assess the feasibility of using ultrasound imaging for this purpose may be useful in future studies.

### Radiotherapy

Radiotherapy is believed to reduce the development of myofibroblasts [14, 52], although the precise mechanism of action remains unclear [60]. A number of publications reporting radiotherapy treatment for early DD could not be included for review as it was not possible to extract data for early DD patients from the total cohort according to the review criteria and studies including patients who had previously received other interventions (see Additional file 2).

Ten radiotherapy studies met the inclusion criteria. The studies were limited by a lack of quality, with no blinding or randomization and the use of subjective outcome measures. Participant numbers were small (10 or less) in 4 of the 10 studies [50–53]. Of the remaining 6 studies 2 reported improvement [14, 45], 3 were equivocal [46, 47, 49] and 3 showed no change [48]. Weinzierl noted that results in his study did not differ clearly from the natural history of early DD [49].

Toxicity should be considered when using radiotherapy for a benign disorder. Toxicity was recorded according to European Organization Research and Treatment of Cancer (EORTC) criteria [61] by one study [14] or LENT-SOMA criteria [47], although sub analysis according to the grade of DD was not reported. At a mean follow-up of 6 years (range 1–12) of the total cohort of 142 hands (96 patients) reported by Keilholz, 61 hands (43 %) developed grade 1 reactions and 14 Grade 2 reactions with pronounced erythema and moderate edema [14]. No Grade 3 or 4 reactions were observed. Over a median 10 year follow up (range 7–18 years), 44 hands developed significant desquamation and 15 subcutaneous atrophy and telangiectasia [47]. Six further studies reported acute and long term effects. Toxicity was not reported in 2 studies [45, 48]. In the UK, The National Institute for Health and Care Excellence (NICE) advises that there is limited evidence regarding the safety of radiation therapy for early DD but does not raise any serious safety concerns. However, there is a theoretical risk that patients could develop radiation induced cancer in the long term [60].

There were inconsistencies in the definition of early disease in studies reporting the efficacy of radiotherapy. Early disease was defined as without contractures in one study [45], whereas data for patients with Tubiana stages 2 and 3 i.e. a total flexion contracture of between 45 and 135°, were included in other studies of early DD [14, 47], although it was possible to separately analyse the data for patients with less severe contractures.

### Collagenase

The use of clostridial hystolyticum collagenase injection was not included in the review as the Food and Drug Administration (FDA) approval [62] was given for use in adult patients with advanced DD. More recent studies reporting results of collagenase injection have included patients with early disease as defined as a palpable cord with up to 30° of digital contracture [63–68]. However, the role of collagenase in early DD has been questioned as the safety and efficacy of collagenase injection for early disease was not included in the original submission to the FDA that led to the approval of collagenase flexion deformities greater than 20° [69].

### Limitations of the review

Identifying the efficacy and safety of non-surgical treatments for patients with early DD in this review was challenging due to the poor quality of studies, small numbers of participants in many studies and the variable definitions of early disease and disease progression. Additionally, limited details on study methodology made evaluation difficult.

Eleven of the 19 case series described results for less than 10 early DD patients [18, 33, 34, 40–42, 44, 50–53] and are unlikely to have been adequately powered to permit conclusions. Only 4 studies described results for cohorts of early DD patients [31, 46, 48, 49] as defined by the criteria of this review. The remaining studies included early DD patients within cohorts of patients with more advanced disease or were case studies.

The search terms were selected to be inclusive but it is possible that some studies were not found by our review. The use of Tubiana grading to define the severity of DD by some studies resulted in the inclusion of patients with total digital contractures of up to 45° in the analysis. It could be argued that this represents patients with relatively advanced DD. However the total digital flexion calculation used by Tubiana classification could relate to 2 or 3 mildly contracted joints, although it could equally apply to 1 more severely affected joint, with other joints unaffected. This highlights the problems associated with this type of grading.

Clinical assessment of size and consistency of nodules and cords was used as an outcome measure in a number of studies [14, 30–34, 45, 46, 48–51]. However, it may be difficult to ascribe nodule resolution to treatment as nodules have been reported to regress spontaneously. An epidemiological study conducted in Iceland reported that of 56 men initially noted to have nodules or cords, 8 were judged to have normal hands 18 years later [5]. The variation in diagnostic criteria for DD has been noted [3, 70]. It has been suggested that 2 types of DD exist, typical (progressive) and atypical (non-progressive), with typical disease often progressing to require surgical intervention whilst treatment is rarely indicated for atypical DD [71]. Each type may produce a different response to treatment. More recently fluctuation in the course of early DD has raised uncertainty about the natural history of DD [7].

## Conclusions

Given the limitations of treatments available for advanced digital contractures, the ideal treatment for patients with progressive DD would be at the early stage to prevent the development of flexion deformities [8]. Of the many treatments that have been tried over the years for early DD, only intralesional steroid injection or radiotherapy appear to offer some benefit. The studies reporting the effect of Intralesional steroids were confounded by the lack of a control group and potential assessor bias. The literature is divided on the efficacy of radiotherapy and effect on the course of early DD with some showing it is efficacious but others demonstrating little or no benefit. Unfortunately there is lack of objective evidence for efficacy of either intralesional steroid injection or radiotherapy and there is an urgent need for adequately powered double blinded randomized trials. Disease recurrence following treatment is infrequently reported in the reviewed studies. It is not clear how long recurrence should be monitored but a study of intralesional steroid injection [31] suggests that this may need to be in excess of 3 years.

For future studies investigating the effectiveness of non-surgical treatments of early DD, we would recommend the following:

- A clear definition of early DD and a consensus on a definition of disease recurrence is also essential to allow comparison between studies.
- All treatment outcomes should be measured using objective, reproducible methods, including:
  1. Goniometric measurement of extension and flexion of individual joints.
  2. A reliable and validated measure of nodule consistency and the role of tonometry, which has been used to assess Dupuytren’s pre-and post-surgery [72], should be investigated.
  3. The use of ultrasound imaging to monitor change in nodule size.
  4. New disease specific Patient reported outcome measures (PROMs). These need to be developed to gain the patient’s perspective in early disease and to monitor disease progression. PROMS have been developed and validated in advanced disease but these are unlikely to reflect the problems of patients with nodules with little or no contracture.
- Studies should be well designed and adequately powered.
- Safety should be reported and described in all studies.

## Abbreviations

DD, Dupuytren’s disease; EORTC, European Organization for Research and Treatment of Cancer; FDA, Food and Drug Administration; GRADE, Grades of Recommendation, Assessment, Development and Evaluation; Gy, Grays; LENT-SOMA, Late Effects Normal Tissue-Subjective Objective Management Analytic; Mg/mgs milligram/milligrams; NICE, National Institute for Health and Care Excellence; OCEBM, Oxford Centre for Evidence Based Medicine; PICOS, Participants Intervention Comparison Outcomes Setting; PRISMA, Preferred Reporting Items for Systematic Reviews and Meta-Analyses; PROMS, Patient Reported Outcomes Measures; PROSPERO, Prospective register of systematic reviews

## Additional files

Additional file 1: Search strategy and Search terms using PICOS analysis. (DOC 31 kb) Additional file 2: Table of excluded studies detailing study design, treatment, numbers of patients and reasons for exclusion. (DOCX 46 kb) Additional file 3: Study eligibility screening sheet. (DOC 26 kb)
