# Supplementary material for: Systematic review of non-surgical treatments for early dupuytren’s disease
Source: BMC Musculoskelet Disord. 2016 Aug 15;17:345. doi: 10.1186/s12891-016-1200-y (PMC4986253; doi:10.1186/s12891-016-1200-y)
Supplement: Additional file 1: — Search strategy and Search terms using PICOS analysis. (DOC 31 kb) [file 12891_2016_1200_MOESM1_ESM.doc]

## Additional file 1

Search strategy and Search terms using PICOS analysis

|  | **Definition** | **Main Search Terms for Ovid Medline and Embase Strategy**  **Subject heading (/) and free text terms** |
| --- | --- | --- |
| **Participants** | Persons with early Dupuytren’s Disease | Dupuytren’s contracture/ or dupuytren |
| **Intervention** | Non-surgical treatment including drug therapy, physical therapy and radiotherapy for early DD of the hand. | Exp steroids/ or exp glucocorticoids/ or dimethyl sulfoxide/ or dimethyl sul??oxide or vitamin e/ or vitamin e or allopurinol or interferon-gamma/ or interferon or fluorouracil or colchine or exp hydroxymethylglutaryl-CoA reductase Inhibitors/ or statin or exp radiotherapy or exp radiation/ or radiother or radiation or ultrasonic therapy/ or ultraso or high energy shock waves/ or shock wave or hyperbaric oxygenation/ or hyperbaric oxygen or (therapy or drug therapy or radiotherapy as floating subheadings) |
| **Comparisons** | Not applicable |  |
| **Outcomes** | Patient reported outcome measures (PROMs), physical measures, functional tests, clinical assessment and clinical observation | As reported in the study. |
| **Study design** | All Included:-  Screen search results manually to include RCT’s and non-randomised controlled clinical trials, prospective and retrospective case series, case studies, conference papers, abstracts and letters. | As described in the study |
