# Supplementary material for: Systematic review of non-surgical treatments for early dupuytren’s disease
Source: BMC Musculoskelet Disord. 2016 Aug 15;17:345. doi: 10.1186/s12891-016-1200-y (PMC4986253; doi:10.1186/s12891-016-1200-y)
Supplement: Additional file 2: — Table of excluded studies detailing study design, treatment, numbers of patients and reasons for exclusion. (DOCX 46 kb) [file 12891_2016_1200_MOESM2_ESM.docx]

| **Author, Title, Journal, Year** | **Type and language of publication** | **Treatment**  (n/a = not applicable) | **Reason for exclusion** |
| --- | --- | --- | --- |
| Aaron, C. and M. J. Quesnel (1978). "Celestene use by ionization for rheumatologic diseases treatment. [French]." Semaine des Hopitaux **54**(33-36): 1091-1094. | Case studies  French | Betamethasone (glucocorticoid) by ionisation | Unable to determine if any of 9 cases had early DD. No baseline data. |
| Al-Qattan, MM, Ketchum, LD. The injection of nodules of Dupuytren's disease with triamcinalone acetonide [2]. Journal of Hand Surgery 2001 26 (3):560-561. | Letter and reply  English | n/a | Letter to the Editor. No data. |
| Aron, E. (1968). "Medical treatment of Dupuytren's disease with a cytostatic agent (methylhydrazine). [French]." La Presse medicale **76**(41): 1956. | Case series  French | Injections of 100mg methylhydrazine 3 days a week for 2 weeks. | Unable to determine if any of 8 patients had early DD although reported to most effective in early cases. More recently reported to be very toxic by skin absorption and causes skin irritation (United States Environmental Protection Agency website 2015) |
| Aron, E. (1977). "Medical treatment of the Dupuytren, Ledderhose and Peyronie diseases with procarbazine (Natulan). [French]." Revue de Medecine de Tours 11(7): 1019-1022. | Case studies  French | Oral Natulan (procarbazine) (antineoplastic) | One case with DD. Not early disease. |
| Baker, DE. New drugs approved by the FDA; New dosage forms and indications approved by the FDA; Agents pending FDA approval; New drug/biologics license applications filed by manufacturer; Significant labelling changes or "dear health care professional" letters related to safety. Hospital Pharmacy October 2009 44 (10):908-912. | Report  English | n/a | Current Drug information. No data. |
| Betz, N, Ott, OJ, Adamietz, B, Sauer, R, Fietkau, R, Keilholz, L. Radiotherapy in early-stage dupuytren's contracture long-term results after 13 years. Strahlentherapie und Onkologie February 2010 186 (2):82-90. | Case series  English | Radiotherapy course of 30 Gy comprising of 5 daily treatments of 3 Gy repeated after 6 to 8 weeks. | 9 pts from total cohort of 135 pts (198 hands) had recurrent disease and received previous treatment for DD. Unable to extract data for those with early DD and no previous treatment. |
| Bray, E, Galeazzi, M. First results in the treatment of Dupuytren's disease. Arthritis and Rheumatism Dec 1980 23 (12):1408. | Letter  English | Lathyrogene administered by electroionophoresis 20 applications once daily each lasting 15 seconds of 2-6 watt intensity of 5 ml on palmar aspect of the hand and on the negative pole. Treatment repeated every 3 months. | Unable to identify whether patients with early DD included within the cohort of 21. |
| Buchan, JF. Heat therapy and ultrasonics. The Practitioner Jan 1972 208 (243):125-131. | Narrative review  English | Ultrasound for DD. | Comments on use of ultrasound for DD. No data. |
| Butturini, U. (1950). "Treatment of Dupuytren's disease with alpha-tocopherol acetate. [Polyglot]." Minerva medica **41**(65): 1235-1237. | Case studies  Italian | Vitamin E oral | 6 case studies. Stage 2 to 3: 3 cases, Stage 3: 2 cases and stage not stated: 1 case. Unable to determine if 1 case had early DD. |
| Buturlina, V. F. (1971). "Effectiveness of treatment of Dupuytren's contracture by ultrasonic therapy in combination with lidase injections. [Russian]." Voprosy kurortologii, fizioterapii, i lechebnoi fizicheskoi kultury **36**(5): 461. | Case series  Russian | Lidase injections and ultrasound | No sub-analysis for 6 cases with baseline Stage 1 early DD from total cohort of 28. |
| Castle, WM. Drugs and fibrotic reactions - Part I. Adverse Drug Reaction Bulletin 1985 NO. 113:420-423. | Bulletin  English | n/a | Drug reaction bulletin. No data. |
| Castle, WM. Drugs and fibrotic reactions. Part II. Adverse Drug Reaction Bulletin 1985 NO. 114:424-427. | Bulletin  English | n/a | Drug reaction bulletin. No data. |
| Chang, J, Longaker, MT. 5-Fluorouracil selectively inhibits collagen synthesis. Plastic and Reconstructive Surgery July 2005 116 (1):222-223. | Scientific paper  English | n/a | Discussion of laboratory findings. No clinical data. |
| Cimmino, MA, Cutolo, M, Beltrame, F. Local injections of tiopronin in Dupuytren's contracture. Arthritis and Rheumatism 1982 25 (12):1505. | Letter  English | Injection of weekly 50 mg tiopronin into palmar apponeurosis close to nodules for 6 months. | Unable to identify whether patients with early DD included within total cohort of 10. |
| Conway, H. Dupuytren's contracture. American journal of surgery Jan 1954 87 (1):101-119. | Narrative review  English | n/a | Review. Non-operative treatment mentioned but not specified. No data. |
| Crisp, EJ, Kendall, PH. Hydrocortisone in lesions of soft tissue. The Lancet 1955 265 (2862): 476-9. | Case reports  English | Intralesional injection of hydrocortisone acetate 25 mg in 1 ml suspension. | Early DD not defined for 4 of 6 patients described as having early DD. No baseline data. |
| De Haas, W. H. (1979). "[Dupuytren's disease. Treatment by local injections with hyaluronidase using a Dermojet]." Acta Rhumatologica **3**(4): 275-285 | Case series  French | Injected hyaluronidase. | Cohort of 38 patients unable or unwilling to have surgery. Not early DD. |
| Di Martino, A, Costa, V, Martinelli, N, Zagami, MG, Denaro, V. Off-label use of MESNA in Dupuytren's disease. Medical Hypotheses 2008 70 (3):702-703. | Letter  English | Intranodular injection of sodium-2-mercaptoethanesulfonate (MESNA). | Hypothesises use of MESNA. No data. |
| Dominguez-Malagon, HR, Alfeiran-Ruiz, A, Chavarria-Xicotencatl, P, Duran- Hernandez, MS. Clinical and cellular effects of colchicine in fibromatosis. Cancer 1992 69 (10):2478-2483. | Case reports  English | Case study. Oral colchicine 3 mg daily for 2 weeks 1 mg for 3 months for patient with DD with limited digital movement due to severe contracture. | Not early DD. |
| English, C, Coughlan, R, Carey, J, Bergin, D. Plantar and palmar fibromatosis: Characteristic imaging features and role of MRI in clinical management. Rheumatology (United Kingdom) June 2012 51 (6) (pp 1134-1136)(ker522). | Letter  English | Case study of role of MRI. | Not early DD. |
| Foldi, M. (1954). "A new hyaluronidase-novocaine infiltration treatment of Dupuytren's disease, A dupuytren-kor uj kezelese hyaluronidase-novocain-infiltratioval. []." Orvosi hetilap **95**(7): 182. | Case study  Hungarian | Hyaluronidase-novocaine infiltration. | Not early DD. |
| Franchini, C. (1952). "A case of Dupuytren's disease treated with vitamin E, Su di un caso di malattia di Dupuytren trattato con vitamina E. []." Rassegna di studi psichiatrici **41**(1): 59-66. | Case study  Italian | Vitamin E (tocopherol) 250mgs daily for 20 days initially, reducing dose to 100mgs then 20mgs | Not early DD. |
| Freeman, AG. Topical treatment for Dupuytren's contracture [2]. Lancet 1995 345 (8946):382. | Letter  English | Topical 0.05% clobetasol proprionate and 0.05% tretinoin cream for 17 months in DD case study. | Not early DD. |
| Garcia,T, de Torres, M, Ludena, B, Rodriguez, A, Caballero, B, Sotoca, A, Caballero, P, Lopez, A, Martin,G. Radiotherapy in Dupuytren's contracture: A single institution's experience. Reports of Practical Oncology and Radiotherapy. 2013:18 Supplement 1, S289-S305 | Conference abstract  English | Radiotherapy course of 21 Gy comprising of 5 daily treatments of 3 Gy. | 4 early DD patients within cohort of 5 had previous surgery. Unable to identify results for remaining early DD patient. Dosage discrepancy in abstract. |
| Herbst, M. and G. Regler (1985). "Dupuytren contracture. Radiotherapy of early stages. [German]." Strahlentherapie **161**(3): 143-147. | Case series  German | Radiotherapy 5 x 3 Gy per week in 2 series totalling 30 Gy | Unable to extract data for 46 patients with early DD from total cohort of 51 |
| Horvath, G. and P. Cisar (1981). "Physical therapy of Dupuytren's contracture. [Slovak]." Fysiatricky a Revmatologicky Vestnik **59**(3): 121-125. | Case series  Slovakian | Radiotherapy | 170 patients contacted at 5 years after treatment, 67 responded. Unable to extract results for patients with early DD. |
| Hueston, JT. Enzymic fasciotomy. The Hand Mar 1971 3 (1):38-40. | Case series  English | Enzymatic fasciotomy. Injection with 2.5 mg trypsin with local anaesthetic and hyaluronidase injected at points along the cord and into nodule. | Unable to identify whether patients with early DD included within the cohort of 12. |
| Hussar, DA, Daniels, WL. New drugs: Sipuleucel-T, cabazitaxel, and collagenase clostridium histolyticum. Journal of the American Pharmacists Association : JAPhA 2010 50 (6):772-775. | Drug Report  English | n/a | Report of new drugs. No data. |
| Keilholz, L., et al. (1997). "[Radiotherapy in the early stage of Dupuytren's disease. The indications, technic and long-term results]." Strahlentherapie und Onkologie **173**(1): 27-35. | Case series  German | Radiotherapy 2 courses of 5x3 Gy total 30 Gy with 6 week interval between courses. | Duplicate data. German version of included Keilholz 1996 publication. |
| Ketchum, LD. The rationale for treating the nodule in Dupuytren's disease. Plastic and Reconstructive surgery Global Open 2014;2:e278 | Narrative review  English | n/a | Review. No data. |
| King, RA. Vitamin E therapy in Dupuytren's contracture; examination of the claim that vitamin therapy is successful. Journal of Bone & Joint Surgery - British Volume Aug 1949 31B(3):443. | Short report  English | Oral vitamin E 100 mg alpha-tocopherol 3 times daily for up to 8 weeks. | Unable to identify whether patients with early DD included within the cohort of 13. |
| Kirk, JE, Chieffi, M. Tocopherol administration to patients with Dupuytren's contracture; effect on plasma tocopherol levels and degree of contracture. Proceedings of the Society for Experimental Biology and Medicine 1952 1952 Society for Experimental Biology and Medicine (New York, N.Y.). 80 (4):565-568. | Conference proceedings report  English | Oral vitamin E 300 mg alpha-tocopherol daily for 300 days. | Unable to identify data for early DD patients in total cohort of 19. |
| Langston, RG, Badre, EJ. Dupuytren's contracture. Canadian Medical Association Journal 1948 58(57-61) | Discussion paper  English | Reports use of Vitamin E (tocopherol). Dose as recommended by Steinberg. | Unable to identify whether patients with early DD included within the cohort of 5. |
| Leibel, D, Wong, J, Jackson, T. The use of electron radiation therapy to treat early stage dupuytren's contracture. Journal of Medical Imaging and Radiation Sciences March 2012 Conference: 17th World Congress of the International Society of Radiographers and Radiological Technologists, ISRRT and the 70th Annual General Conference of the Canadian Association of Medical Radiation Technologists, CAMRT Toronto, ON Canada. Conference Start: 20120607 Conference End: 20120610. Conference Publication: (var.pagings). 43 (1 SUPPL. 1):S57. | Conference abstract: poster  English | Radiation therapy course of 30 Gy delivered in 10 fractions. | Abstract contains no data. |
| Lopater, Z, Jones, DA, Dusenbery, K. Treatment of dupuytren contracture and ledderhose disease with radiation. International Journal of Radiation Oncology Biology Physics 01 Sep 2014 1):S684. | Abstract  English | Radiotherapy total dose 30 Gy comprising 5 daily fractions of 15 Gy repeated after 4-6 weeks. | Included feet as well as hands. Unable to extract data for early DD patients. |
| Mazzoni, G. (1954). "A case of acute nodose rheumatism with Dupuytren's contracture treated with cortisone. [Italian]." Reumatismo **6**(6): 392-397. | Case study  Italian | Oral cortisone 100mgs per day for 5 days | Case study of acute rheumatism with Dupuytren’s type appearance of the hands. Unable to determine if DD and stage of disease. |
| McCarthy, DM. The long-term results of enzymic fasciotomy. Journal of Hand Surgery 1992 17 B (3):356. | Case series  English | Enzymatic fasciotomy. Injection with 2.5 mg trypsin with local anaesthetic and hyaluronidase. | Unable to identify whether patients with early DD included within the cohort of 14. |
| Meletis, CD, Barker, JE. Therapeutic enzymes: Using the body's helpers as healers. Alternative and Complementary Therapies 2005 11 (2):74-77. | Narrative review  English | n/a | Reviews use of enzymes for different condition. No data. |
| Millesi, H. (1965). "[On the pathogenesis and therapy of Dupuytren's contracture. (A study based on more than 500 cases)]." Ergebnisse der Chirurgie und Orthopadie **47**: 51-101. | Narrative review  German | n/a | Narrative review within book chapter of non-operative treatment but disease stage not specified. |
| Montero Luis, A, Hernanz de Lucas, R, Hervas Moron, A, Fernandez Lizarbe, E, Sancho Garcia, S, Vallejo Ocana, C, Polo Rubio, A, Ramos Aguerri, A. Radiation therapy for the treatment of benign vascular, skeletal and soft tissue diseases. Clinical and Translational Oncology 2008 10 (6):334-346. | Review  English | n/a | Review of use of RT for DD. No data. |
| Mullard, A. 2010 FDA drug approvals. Nature Reviews Drug Discovery February 2011 10 (2):82-85. | Report of drug approvals  English | n/a | Report of new drug approvals. No data. |
| Mundanthanam, GJ, Rozental, TD. Journal scan: Journal of hand surgery. Clinical Orthopaedics and Related Research February 2010 468 (2):631-636. | Summary  English | n/a | Summary of articles in Journal of Hand Surgery. No data. |
| Murrell, GAC, Hooper, G. An insight into Dupuytren's contracture. Annals of the Royal College of Surgeons of England 1992 74 (3):156-161. | Commentary  English | n/a | Narrative review. No data. |
| Namazi, H, Abdinejad, F. Botulinum Toxin as a novel addition to the antidupuytren armamentarium. Medical Hypotheses 2007 68(1):240- | Letter  English | n/a | Hypothesises about subcutaneous injection of botulinum toxin |
| Nedelka, MA, Weiss, E. Radiation therapy for early stage palmar and plantar fibromatosis: A treatment option for patients who wish to avoid disease progression and surgery. International Journal of Radiation Oncology Biology Physics 01 Oct 2013 1):S561. | Conference abstract: poster  English | Radiotherapy course of 30 Gy comprising 10 fraction split midcourse by 6-8 weeks or a total of 21 Gy in 7 daily fractions with no break. | Unable to identify patients with early DD within cohort of 16 patients (19 hands, 3 feet). |
| Nikolova, L. (1962). "Treatment of Dupuytren's contracture with ultrasonics and paraffin application. [Bulgarian]." Khirurgiia **15**: 1097-1100. | Case series  Bulgarian | Paraffin hand baths followed by ultrasound treatment. | 25 pts had no previous treatment, 7 had other treatment (unspecified) and 3 had 3 previous treatments (unspecified). Unable to extract data for pts with early DD who had not received previous treatments. |
| Palchun, V. T., et al. (1995). "Intravascular laser irradiation of the blood in the treatment of suppurative septic complications in otorhinolaryngology. [Russian]." Vestnik otorinolaringologii(2): 8-10. | Case reports  Russian |  | 5 patients with Dupuytren’s phlegm mentioned in abstract. Throat disorder discussed in the text but not Dupuytren’s disease of the hand. |
| Parsons, AR. Dupuytren's contracture; treatment by massive doses of vitamin E. Irish journal of medical science 1948 19 (270):272-274. | Case Report  English | Oral Vitamin E 20 mg 3 times daily gradually increasing to 300 mg daily and continued for over 100 days. | Unable to identify if patient had early DD. |
| Pastremoli, A. (1969). "Treatment of Dupuytren's isease by superficial roentgentherapy with a beryllium-window tube. [Italian]." The Journal of communication **19**(3): 142-157. | Publication unable to be accessed  Italian | Radiotherapy | British Library unable to obtain a copy of the manuscript. Probably incorrectly referenced. |
| Pittet. Effect of gamma-interferon on the clinical and biologic evolution of hypertrophic scars and Dupuytren's disease: an open pilot study. Plastic & Reconstructive Surgery May 1994 93(6):1224-35. | Case series  English | Intralesional injection of 200ug (0.2 mg) y-interferon 2 times weekly for 4 weeks with oral premedication of acetominophen. | Included patients with Ledderhose disease. Unable to identify results for patients with early DD within total cohort of 7. |
| Pitts Jr, FN. Colchicine therapy for palmar fibromatosis [7]. New England Journal of Medicine 1995 333 (6):393. | Letter: case report  English | 1.2 mg colchicine daily for 7 days increasing to 0.6 mg daily for 3 weeks then 1.2 mg twice weekly for 2 years. | Unable to identify if patient had early DD. |
| Pohl S, Hinke A, Attassi M, Guntrum F, Seegenschmiedt HM. Prophylactic radiotherapy in Dupuytren's contracture: Three years outcome. International Journal of Radiation Oncology Biology Physics 54: 23 | Abstract  English | Group A: radiotherapy course of 30 Gy comprising 5 daily treatments of 3 Gy repeated after 8 weeks. Group B: 21 Gy comprising 7 daily treatments of 3 Gy within 2 weeks. | Cohort included Tubiana stages 2. Unable to extract data for early DD patients. |
| Pospisilova, J, Samohyl, J, Koprivova, M, Jelinkova, A. Our experience with the use of ultrasound in rehabilitation of hand. Acta chirurgiae plasticae 1980 22 (4):191-199. | Case series  English | Ultrasound. | Post-surgical DD patients. |
| Rabito, C., et al. (1975). "Results with griseofulvin in the management of some forms of connectivopathy. [Italian]." Giornale Italiano di Dermatologia / Minerva Dermatologica **110**(11): 597-600. | Case reports  Italian | Griseofulvin | 1 case with later stage DD from cohort of 22 cases. Not early DD. |
| Ritchey, SJ. The use of cortisone in Dupuytren's contracture. United States Armed Forces medical journal Jun 1952 3 (6):811-818. | Case reports  English | n/a | Not early DD. |
| Rosenbaum, EE, Herschler, RJ, Jacob, SW. Dimethyl Sulfoxide in Musculoskeletal Disorders. JAMA Apr 26 1965 192:309-13. | Commentary  English | Topical dimethyl sulfoxide 15 to 30 mls daily for 2 weeks to maximum 3 months treatment. | Unable to identify whether patients with early DD included within the cohort of 3. |
| Saraf, S. Dupuytren's disease. Indian Journal of Dermatology, Venereology and Leprology 01 May 2010 76 (3):291-293. | Letter  English | n/a | Describes case and summarises treatment. No data. |
| Schechtman, AD. Combining triamcinolone and lidocaine for soft tissue injections. American Family Physician 15 May 2008 77 (10):1372; author reply 1372. | Letter  English | n/a | Letter responding to published article. No data. |
| Schuster, JM, Saraiya, S, Nedelka, MA, Weiss, E, Tennyson, N. Patient-reported outcome after electron radiation treatment for early-stage palmar and plantar fibromatosis. International Journal of Radiation Oncology Biology Physics 01 Sep 2014 1):S686-S687. | Abstract: poster  English | Radiotherapy Average dose 26.6 Gy 30 Gy in 10 fractions with 6-8 week break after 15 Gy or 21 Gy in 7 fractions | Included 15 cases of plantar disease and 42 hands. Unable to extract data for early DD patients from total cohort of 35. |
| Seegenschmiedt, HM, Schubert, C, Wielputz, M, Hinke, A. Radiotherapy for early-stage Morbus Dupuytren long-term outcome of a randomized prospective trial. Strahlentherapie und Onkologie June 2010 Conference: 16. Jahreskongress der Deutschen Gesellschaft fur Radioonkologie, DEGRO 2010 Magdeburg Germany. Conference Start: 20100603 Conference End: 20100606. Conference Publication: (var.pagings). 186:44. | Abstract  English | Group A: radiotherapy course of 30 Gy comprising 5 daily treatments of 3 Gy repeated after 6 to 8 weeks. Group B: 21 Gy comprising 7 daily treatments of 3 Gy within 2 weeks. | Included Tubiana stages 2-4. Unable to extract data for early DD patients from cohort of 129 patients. 62 patients had received one or more previous treatments including surgery. |
| Seegenschmiedt, MH, Olschewski, T, Guntrum, F. Radiotherapy optimization in early-stage dupuytren's contracture: First results of a randomized clinical study. International Journal of Radiation Oncology Biology Physics 01 Mar 2001 49 (3):785-798. | Abstract  English | Group A: radiotherapy course of 30 Gy comprising 5 daily treatments of 3 Gy repeated after 6 to 8 weeks. Group B: 21 Gy comprising 7 daily treatments of 3 Gy within 2 weeks. | Included Tubiana stages 2-4. Unable to extract data for early DD patients from cohort of 412 patients. |
| Sommer, AP, Zhu, D. Phototherapy miracles in a nutshell. Photomedicine and Laser Surgery 01 Jun 2009 27 (3):527-528. | Letter  English | Phototherapy 670 nm for advanced DD. | No data. |
| Steinberg, CL. Fibrositis (muscular rheumatism) including Dupuytren's contracture: a new method of treatment. New York State Journal of Medicine Aug 1 1947 47(15):1679-82. | Narrative review  English | n/a | Discusses use of vitamin E. No data. |
| Stiles, PJ. Ultrasonic therapy in Dupuytren's contracture. Journal of Bone & Joint Surgery - British Volume Aug 1966 48(3):452-4. | Case series  English | Ultrasound combined with passive stretching. | Unable to identify data for early DD patients in cohort of 9 patients (13 hands). |
| Sturm, M. and M. Lebeuf (1971). "Results of an immunodepressive treatment in La Peyronie's disease, Dupuytren's disease and certain sclerodermas. [French]." Bulletin de la Societe francaise de dermatologie et de syphiligraphie **78**(5): 523-525. | Case reports  French | Oral Natulan (antineoplastic) | 3 cases with Peyronies disease, one associated with existing DD. No early DD. |
| Thomson, GR. The treatment of Dupuytren's contracture with vitamin E; report of a case. Glasgow Medical Journal Sep 1949 30(9):329-32. | Case report  English | Oral vitamin E 30 mg weekly. | Not early DD |
| Thomson, GR. Treatment of Dupuytren's contracture with vitamin E. British Medical Journal 17 Dec 1949 2 (4641):1382, illust. | Case series  English | Vitamin E (ephynal) four 50 mg tablets daily for minimum 12 weeks up to 20 weeks. | 13 'early' cases (22 hands) had 90 degrees of digital contracture. Did not meet inclusion criteria. |
| Tilley, AR, McDonald, G. Cortisone in treatment of Dupuytren's contracture. Treatment services bulletin Feb 1953 Canada. Dept. of Veterans' Affairs. 8 (2):60-62. | Case series  English | Oral cortisone 400 mg. per day in divided doses decreasing to 200 mg. per day when the eosinophil count showed a satisfactory reduction. | Adjunct to surgery for DD. 7 patients treated either preoperatively or postoperatively or both pre- and post-operatively. Not early DD. |
| Trafton, PG. What's new in orthopaedic surgery. Journal of the American College of Surgeons 2001 193 (6):641-648. | Narrative review  English | n/a | A review. No data. |
| Vuopala, U, Kaipainen, WJ. DMOS in the treatment of Dupuytren's contracture. A therapeutic experiment. Acta rheumatologica Scandinavica 1971 17 (1):61-62. | Case series  English | Topical 80% dimethylsulfoxide 3 times daily for 1 month. Dose not stated. | Unable to identify results for patients with early DD within the cohort of 23 patients. |
| Zirbs, M, Anzeneder, T, Bruckbauer, H, Hofmann, H, Brockow, K, Ring, J, Eberlein, B. Radiotherapy with soft X-rays in Dupuytren's disease- successful, well-tolerated and satisfying. Journal of European Academy of Dermatology and Venereology 2015 May;29(5):904-11. | Retrospective case series.  English | Radiotherapy total dose 32 Gy with an 8 week interval between 4 courses of 2 fractions at 2 consecutive days with a single dose of 4 Gy. | 37 patients had received one or more previous treatments including surgery. Unable to identify early DD patients within cohort of 206 patients. |
