# Supplementary material for: Systematic review of non-surgical treatments for early dupuytren’s disease
Source: BMC Musculoskelet Disord. 2016 Aug 15;17:345. doi: 10.1186/s12891-016-1200-y (PMC4986253; doi:10.1186/s12891-016-1200-y)
Supplement: Additional file 3: — Study eligibility screening sheet. (DOC 26 kb) [file 12891_2016_1200_MOESM3_ESM.doc]

**SCREENING TOOL: STUDY ELIGIBILITY**

**Systematic review of outcomes of nonsurgical treatment for early Dupuytren’s Disease**

Authors ……………………………………………………………………………………………………………………………………….

Title …………………………………………………………………………………………………………………………………………….

Date ……………………………………….. Journal……………………………………………………………………………………..

From reading the Title and Abstract or if abstract unavailable, search headings, please answer the following:

**Q1. Participants**

Have any participants had a diagnosis of Dupuytren’s Disease (DD) of the hand?

YES: Go to question 2

NO: Exclude

UNCLEAR: Refer for discussion

**Q2. Nonsurgical treatment**

Have participants had a nonsurgical treatment for DD? (drugs including topicals, radiotherapy, physical therapies including splinting and therapeutic ultrasound. Excluding collagenase injection or skeletal traction)

YES: Go to question 3

NO: Exclude

UNCLEAR: Refer for discussion

**Q3. Outcomes**

Did the study report outcomes? (Disease status: DD classification stage, nodule and/or cord size, number, disease progression. Physical measures: range of motion, grip and pinch strength, sensation. Patient reported: satisfaction, hand function).

YES: Go to question 4

NO: Exclude

UNCLEAR: Refer for discussion

**Q4. Early Dupuytren’s Disease**

Do subjects have early disease identified by flexion contracture of 30 degrees or less at the MCP or PIP joint, Tubiana stage N, N/1, or 1, Shaw grade 1 or 2? Can the data or descriptor for early disease subjects be identified within the cohort?

YES: Go to question 4

NO: Exclude

UNCLEAR: Refer for discussion

Reviewer (circle) CB/DI/LV/JN Date Decision INCLUDE / EXCLUDE/ REFER
